# HHV8-Negative Primary Effusion Lymphoma of B-Cell Lineage: Two Cases and a Comprehensive Review of the Literature

**DOI:** 10.1155/2013/292301

**Published:** 2013-01-16

**Authors:** Neeraj Saini, Ephraim P. Hochberg, Erica A. Linden, Smita Jha, Heinz K. Grohs, Aliyah R. Sohani

**Affiliations:** ^1^Department of Internal Medicine, North Shore Medical Center, Salem, MA 01970, USA; ^2^Center for Lymphoma, MGH Cancer Center, Massachusetts General Hospital, Boston, MA 02114, USA; ^3^Department of Medicine, Harvard Medical School, Boston, MA 02115, USA; ^4^Department of Pathology, North Shore Medical Center, Salem, MA 01970, USA; ^5^Department of Pathology, Massachusetts General Hospital and Harvard Medical School, Boston, MA 02114, USA

## Abstract

Primary effusion lymphoma (PEL) is a rare extranodal lymphoma that typically presents in a body cavity in the absence of a detectable tumor mass and that occurs predominantly in immunosuppressed individuals. The neoplastic lymphoid cells are frequently infected with human herpes virus 8 (HHV8), also known as Kaposi sarcoma herpes virus (KSHV). We describe two HIV-negative patients who presented with primary effusion lymphoma of B-cell lineage involving the pleural cavity, but whose tumor cells lacked infection by HHV8. We review the English language literature of HHV8-negative PEL of B-cell lineage and compare these lymphomas to HHV8-associated PEL with regard to clinical and pathological characteristics, therapy, and outcome.

## 1. Introduction

Primary effusion lymphoma (PEL) is a rare extranodal lymphoma of large B cells with characteristic clinicopathologic features including: initial presentation as a body cavity lymphomatous effusion in the absence of a detectable tumor mass; occurrence mostly in human immunodeficiency virus (HIV)-positive individuals; and expression of antigens associated with a late stage of B-cell differentiation, such as CD138 and MUM1/IRF4, without pan-B-cell antigen expression [1]. Human herpes virus-8 (HHV8), also known as Kaposi's sarcoma herpes virus (KSHV), is strongly causally related to PEL and its presence has been incorporated as a diagnostic criterion for PEL [2].

Diffuse large B-cell lymphoma (DLBCL) constitutes approximately 30–40% of all non-Hodgkin's lymphoma (NHL) and typically presents with a rapidly enlarging symptomatic mass, usually due to nodal enlargement. Extranodal disease with involvement of tissue other than lymph node, spleen, Waldeyer's ring or thymus is quite common in DLBCL, as is secondary involvement of a body cavity by DLBCL [3]. However, primary presentation of DLBCL as a body cavity lymphomatous effusion without any detectable solid mass, similar to HHV8-associated PEL, is extremely rare. Reports of such cases of HHV8-negative PEL of B-cell lineage are limited to isolated case reports and small series. We report two additional cases of this aggressive extranodal lymphoma that presented as a solitary pleural effusion without other sites of disease at the time of diagnosis. In addition, we perform a comprehensive literature review of similar cases with the aim of further characterizing this unusual lymphoma subtype.

**Case 1**

An 87-year-old HIV-negative Portuguese female with a past medical history of heart failure with preserved ejection fraction (EF = 60%), hypertension, atrial fibrillation, dyslipidemia, and degenerative joint disease was admitted with progressive shortness of breath of two weeks' duration. Complete blood count on admission revealed WBC count of 9600/*μ*L, hemoglobin of 13.7 g/dL, hematocrit of 42.0%, and platelet count of 160,000/*μ*L. Serum total protein and LDH were 6.4 g/dL and 184 IU, respectively. The chest X-ray showed an enlarged cardiac silhouette with bilateral pleural effusions. Thoracocentesis revealed the pleural fluid to be exudative with glucose of 3 mg/dL, protein of 3.5 g/dL, LDH of 1341 U/L and 9600 nucleated cells/*μ*L, of which 5100 were normal-appearing white blood cells (6% neutrophils, 91% lymphocytes, 3% monocytes) and 4500 were malignant-appearing cells.

**Figure 1:**
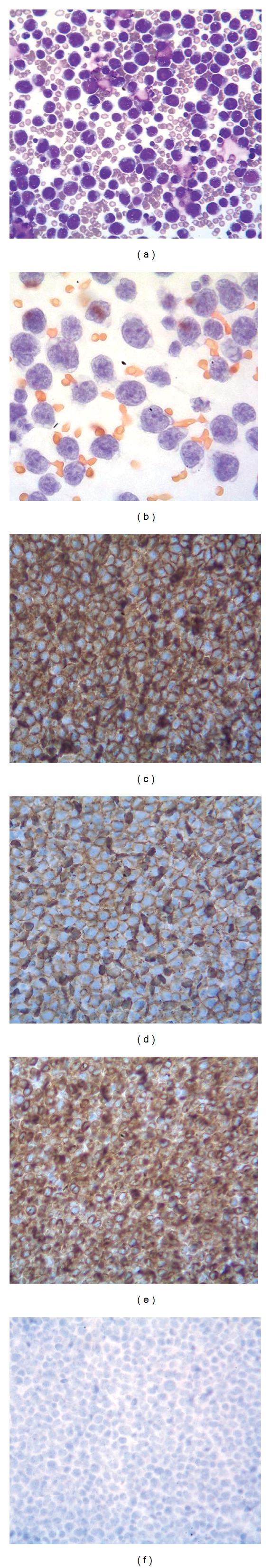
Cytological analysis revealed large atypical lymphoid cells with irregular nuclei, prominent nucleoli and basophilic vacuolated cytoplasm ((a), May-Grünwald-Giemsa; (b), Papanicolaou). By immunohistochemistry of a corresponding cell block specimen, the large cells were strongly positive for CD45 (c), CD20 (d), and CD79a (e), and were negative for CD138 (f), indicative of a mature B-cell immunophenotype.

Cytocentrifuge preparation showed the malignant cells to be large lymphoid cells with irregular nuclei and deeply basophilic cytoplasm with prominent vacuoles (Figures 1(a) and 1(b)). Flow cytometry of the pleural fluid showed that the large cells were positive for CD45, CD19, CD20, CD22, CD79a, CD38, HLA-DR, and surface IgM, with aberrant expression of the T-cell antigen, CD8, and the myeloid antigen, CD13. They were negative for surface and cytoplasmic light chains, MPO, TdT and other T-cell antigens (CD2, CD3, CD4, CD7). Immunoperoxidase stains showed that neoplastic cells were positive for CD45 (Figure 1(c)), CD20 (Figure 1(d)), CD79a (Figure 1(e)), bcl-2, bcl-6 (>50%), Ki-67 (>90%), epithelial membrane antigen (<50%) and negative for CD10, CD30 and CD138 (Figure 1(f)). Immunohistochemical staining for HHV8 latency associated nuclear antigen (LANA)-1 and in-situ hybridization (ISH) for Epstein-Barr virus (EBV) were negative. The patient was diagnosed with DLBCL. Further staging to exclude a primary extra-cavitary site of involvement was performed; however, no mass, organomegaly or lymphadenopathy was detected on computed tomography (CT) scans of the chest, abdomen or pelvis. Ultimately, it was felt that a diagnosis of HHV8-negative PEL was most appropriate. The patient was treated only with talc pleurodesis as she declined chemotherapy and radiotherapy. She is alive approximately 24 months after the procedure and a total of 29 months after her initial presentation of bilateral pleural effusions.

**Case 2**

**Figure 2:**
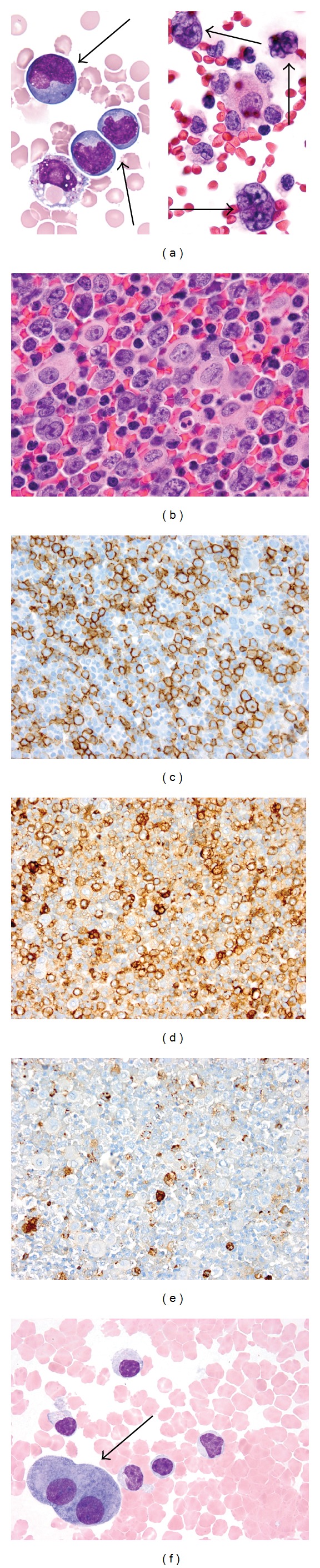
Examination of the initial thoracentesis fluid demonstrated scattered large atypical lymphoid cells with multilobated nuclei, vesicular chromatin and multiple prominent nucleoli (arrows), in a background of benign mesothelial cells, histiocytes, small lymphocytes and neutrophils ((a), left, May-Grünwald-Giemsa, and right, hematoxylin and eosin). The corresponding cell block specimen showed similar findings (hematoxylin and eosin). Immunohistochemical stains showed the scattered large cells to be positive for CD20 (c) and kappa light chain-restricted (d), with few lambda-positive cells in the background (e). A repeat thoracentesis specimen taken 1 month later showed no evidence of malignancy, with only benign mesothelial cells (arrow) and hematopoietic elements (Wright-Giemsa).

An 82-year-old HIV-negative Caucasian female with a past medical history of hypertension, sick sinus syndrome, abdominal aortic aneurysm and chronic obstructive pulmonary disease was admitted with dyspnea. Ten years earlier, she was diagnosed with non-small cell lung cancer that was treated with concurrent neoadjuvant chemotherapy and radiation followed by lobectomy. She had no interval clinical or imaging evidence of recurrence of her thoracic malignancy. Chest radiograph during the admission showed a right-sided pleural effusion. Thoracocentesis revealed malignant cells in the pleural fluid that were large lymphoid cells with irregular nuclear contours, basophilic cytoplasm and multiple nucleoli (Figures 2(a) and 2(b)). Immunohistochemical stains showed the neoplastic cells to be positive for CD20 (Figure 2(c)), PAX5/BSAP, bcl-6, MUM1/IRF4 (subset) and kappa light chain (Figure 2(d)), weakly positive for bcl-2, and negative for CD5, CD10, CD15, CD30, CD138, cyclin D1, lambda light chain (Figure 2(e)) and HHV8 LANA-1. ISH for EBV-encoded RNA (EBER) was negative. Immunoglobulin heavy chain (*IGH@*) gene rearrangement studies showed a clonal pattern [4]. F-Fluorodeoxyglucose (FDG) positron emission tomography (PET)/CT whole body scan for staging did not demonstrate additional sites of disease. The expression of mature B-cell markers and absence of HHV8, EBV, CD30 and CD138 expression excluded the diagnosis of HHV8-associated PEL and the patient was given the diagnosis of HHV8-negative PEL.

The patient became symptomatic with dyspnea a month later and chest X-ray showed recurrent pleural effusion. Thoracocentesis was repeated and examination of the pleural fluid by cytology and flow cytometry revealed only reactive mesothelial cells and histiocytes, without evidence of malignant-appearing cells (Figure 2(f)). No clonal B-cell population was detected by concurrent flow cytometry. Spontaneous regression of lymphoma was re-confirmed with repeat thoracocentesis a week later yielding no malignant cells. However, follow-up FDG PET/CT whole body scans done 4 months later showed a new FDG-avid pleural-based small nodule and various nodularities in the omentum. Tissue biopsy of these nodules was not attempted, but they were believed to be consistent with metastatic progression of the lymphoma. The patient refused any chemotherapy and died 11 months after her diagnosis of lymphoma.

## 2. Design and Methods

“Primary effusion lymphoma” and “body cavity based lymphoma” were used as search terms to identify English-language articles from PubMed published in the past 15 years (January 1997 to June 2012). Primary effusion lymphoma was defined by the presence of malignant lymphoma cells exclusively in a body cavity or cavities without any contiguous or non-contiguous tumor mass or lymph node enlargement at the time of presentation. The review was restricted to reports of primary effusion lymphomas that were negative for HHV8 and that showed expression of mature pan-B-cell antigens. Editorials, reviews without additional cases, and non-published abstracts were excluded.

Clinical information abstracted for each case included: age at presentation; sex; HIV status by enzyme-linked immunosorbent assay (ELISA) or Western blot studies; detection of hepatitis C virus (HCV) by serologic studies or polymerase chain reaction (PCR); detection of EBV by PCR; site(s) of disease; therapy; and outcome. Pathological data collected for each case included: lymphoma cell morphology and immunophenotype; HHV8 LANA-1 expression by immunohistochemistry or detection of HHV8 by PCR or ISH; detection of EBV by EBV latent membrane protein-1 (LMP1) expression or EBER ISH; and results of *IGH@* gene rearrangement and cytogenetic studies.

## 3. Results

The preliminary search for reports using the above mentioned terms yielded 1187 articles. After excluding reports of HHV8-associated PEL and cases of T-cell or null immunophenotype, we identified 34 articles describing 46 unique cases [4–37]. Our review includes these 46 cases and our 2 cases for a total of 48 reported cases of HHV8-negative PEL. 

Clinical characteristics are summarized in Table 1 and detailed clinical and pathological findings in each case are listed in Table 2. The 48 patients had a median age at diagnosis of 74 years (range: 14–99 years) with a male-to-female ratio of 3 : 2. Information regarding HIV status was available in 41 patients, and none were reported to be HIV-positive. The association with HCV and EBV infection was found to be 22.2% and 21.3%, respectively. For the 41 patients with information available regarding site of disease, the frequencies of various sites of involvement were as follows: pleura: 65.9%, peritoneum: 39.0%, and pericardium: 36.6%. A single case (case 48) involved the scrotum.

Most cases consisted of medium-sized to large or large-sized cells that were occasionally described as pleomorphic. All cases expressed one or more pan-B-cell antigens (CD19, CD20 and/or CD79a) and several cases expressed surface and/or cytoplasmic immunoglobulin, antigens typically absent in HHV8-associated PEL [16]. The immunophenotype was variable with regard to germinal center (CD10, bcl-6) and post-germinal center (MUM1/IRF4) markers, but no case was reported to express CD138, a plasmacytic antigen typically seen in HHV8-associated PEL. Expression of T-cell antigens, a feature reported in occasional cases of HHV8-associated PEL, was seen in only rare cases (two cases with CD5 co-expression [22, 23] and case 1 reported above with CD8 co-expression). At least some cytogenetic information (FISH and/or karyotype) was available in 26 cases. Of these, 12 cases showed a rearrangement or amplification involving *MYC* at 8q24 and 13 were reported to harbor a complex karyotype, although full karyotypic information was available in only a small number of cases.

Thirty patients (62.5%) received chemotherapy with a variety of regimens, including cyclophosphamide, doxorubicin, vincristine, prednisolone (CHOP) in 11 patients; CHOP with rituximab (R) in 3 patients; THP-CVP (pirarubicin, cyclophosphamide, vincristine, prednisolone) in 6 patients; THP-CVP + R in 4 patients; and other chemotherapy regimens in 6 patients. Treatment was unknown in 1 patient. The remaining 17 patients (35.4%) received no chemotherapy and were treated with fluid drainage alone or fluid drainage and pleurodesis. The median overall survival (OS) was 11 months. The number of patients alive at 6 months and 1 year following symptomatic presentation was 77.8% and 61.1% respectively. Patients who received no chemotherapy had a median OS of 8 months (range: 1 week to 80 months) versus 12 months (range: 18 days to 38 months) in patients who received any kind of chemotherapy. The rate of death with any kind of chemotherapy at 6 months was 20% and at 1 year was 33%, compared to 25% and 42% without any chemotherapy. Among the 22 patients who died of their lymphoma, the median OS was 7 months (range: 1 week to 80 months). The median follow-up period in 24 living patients was approximately 14.5 months (range: 2 months to 55 months).

## 4. Discussion

**Figure 3:**
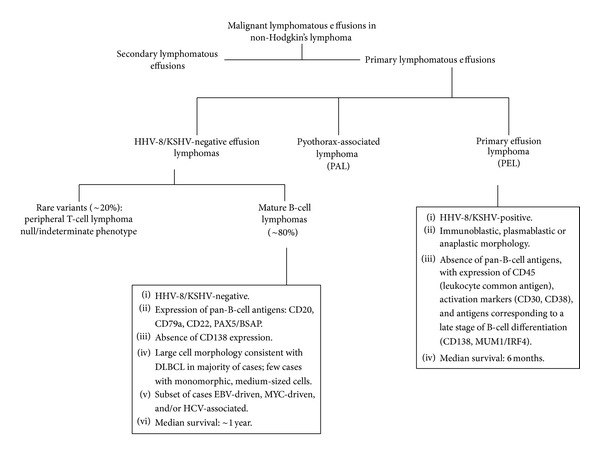
A schema of malignant lymphomatous effusions in non-Hodgkin's lymphoma highlighting the differences between HHV8-associated PEL and HHV8-negative PEL.

Body cavity-based lymphomas are a heterogeneous group of rare non-Hodgkin's lymphomas that arise primarily in the serous body cavities and that result in recurrent effusions. This group includes pyothorax-associated lymphoma and PEL. Pyothorax-associated lymphoma presents as a solid mass localized in the thoracic cavity that is contiguous with the effusion; it is EBV-associated and arises in the setting of long-standing pyothorax resulting from iatrogenic pneumothorax used to treat tuberculosis [38]. In contrast, PEL is typically confined to a body cavity and grows in a liquid phase, without any detectable nodal or extranodal mass elsewhere in the body. As illustrated in Figure 3, PEL can be broadly divided into two categories: HHV8-associated PEL, a subtype of DLBCL and a distinct category in the *2008 WHO Classification of Neoplasms of Haematopoietic and Lymphoid Tissues *[2], and HHV8-negative PEL, of which the majority (~80% of cases) express mature pan-B-cell antigens [39]. As only rare cases of HHV8-negative PEL with neoplastic cells of T-cell derivation [40–44] or null/indeterminate immunophenotype [22, 45, 46] have been reported, we restricted our review to HHV8-negative PEL of B-cell lineage. Our report of two cases and review of the literature demonstrates that these neoplasms are heterogenous, but share common features that distinguish them from HHV8-associated PEL with regard to pathogenesis, clinical characteristics, immunophenotype and prognosis. 

While none of the 48 patients with HHV8-negative PEL were HIV-positive, EBV infection was seen in 21.3% of cases, suggesting that altered immunosurveillance may play a pathogenetic role in some cases. Among the 10 EBV-positive cases, all patients were >50 years old, 2 patients had idiopathic CD4+ T-cell lymphopenia and 1 patient had a history of common variable immunodeficiency, supporting this hypothesis [47]. Similarly, 22.2% of patients had underlying HCV infection. HCV has been shown to be lymphotropic and has been implicated as an etiological factor in lymphomagenesis for various NHL subtypes [48, 49]. Ascoli et al. detected HCV RNA in the ascitic fluid of a patient with HHV8-negative PEL and proposed that HCV may play a causative role in these lymphomas by chronic stimulation of B cells followed by clonal expansion [16].

In our review, a number of cases were negative for both EBV and HCV (19/48; 39.6%), suggesting that other etiologies also play a role in lymphomagenesis. Among cases with available cytogenetic information, 50% (12/26) harbored amplification or rearrangement of the *MYC* oncogene, a likely driver of neoplasia in these cases. In terms of B-cell NHL subclassification, most *MYC*-rearranged cases had large cell morphology or a complex karyotype, consistent with DLBCL [14, 19, 22, 23, 26, 31, 50] Two cases (cases 17 and 18) reportedly had monomorphic medium-sized cells, a germinal center immunophenotype (expression of CD10), and a relatively simple background karyotype, suggesting that these cases may represent an unusual extranodal presentation of Burkitt's lymphoma [16, 17].

Unlike HHV8-associated PEL, HHV8-negative PEL of B-cell lineage shares several clinical features with nodal or extranodal DLBCL presenting with a mass lesion, and differentiating HHV8-negative PEL from conventional DLBCL complicated by secondary lymphomatous effusion requires a thorough staging evaluation to exclude the presence of a mass lesion at the time of diagnosis. Based on our literature review, HHV8-negative PEL presents at an older median age (74 years) compared to that reported for HHV8-associated PEL (44 years) and exhibits a lower male-to-female ratio, similar to DLBCL [2, 44, 51, 52]. The overall favorable prognosis compared to HHV8-associated PEL was underscored by a survival rate of approximately 60% at 1 year and a median OS of 11 months. While this compares favorably to HHV8-associated PEL with a reported median OS of 4–6 months [2, 44], the range was quite wide (1 week to 80 months). This heterogeneity in clinical behavior is further highlighted by our finding of a small number of patients who presented with involvement limited to a single body cavity site, but who developed mass lesions outside of the body cavity at the time of disease progression, similar to our case 2 [32]. At the other extreme, our case 1 showed regression of the malignancy after pleurodesis and drainage of the pleural fluid without any chemotherapy, and in our literature review we identified 7 other patients in whom the lymphoma similarly regressed following drainage of the effusion [5–7, 12, 18, 23]. 

There is no clear consensus on the appropriate treatment of HHV8-negative PEL due to the limited number of cases reported. Our findings suggest that chemotherapy benefits most patients, as those treated with any type of chemotherapy overall had a lower rate of death compared to patients who received no chemotherapy. Remarkably, the addition of rituximab to chemotherapy regimens induced remission in all 8 patients, 7 of whom were alive at the time of last follow-up [4, 8, 12, 13, 29, 31, 32]. The single patient who died following a rituximab-containing regimen died prematurely of a cause unrelated to lymphoma [8]. Therefore, treatment with drainage of the effusion followed by chemoimmunotherapy with rituximab and CHOP, particularly in CD20-positive cases, appears to offer the possibility of prolonged survival in a subset of patients. Further study of rare patients who undergo spontaneous regression of their lymphoma following drainage alone may help to identify clinical or pathological features that predict for a good outcome following only minimal therapy.

## Figures and Tables

**Table 1:** Summary of clinical characteristics of 48 patients with HHV8-negative effusion lymphomas of B-cell lineage.

Characteristics	Number of patients (%)
Age (*n* = 48)	
Age > 60	10 (20.8)
Age < 60	38 (79.2)
Sex (*n* = 48)	
Male	29 (60.4)
Female	19 (39.6)
EBV status (*n* = 47)	
Positive	10 (21.3)
Negative	37 (78.7)
HCV status (*n* = 36)	
Positive	8 (22.2)
Negative	28 (77.8)
Site(s) involved (*n* = 41)	
Pleura	27 (65.9)
Peritoneum	16 (39.0)
Pericardium	15 (36.6)
Treatment (*n* = 48)	
No chemotherapy	17 (35.42)
CHOP	11 (22.92)
CHOP + R	3 (6.25)
THP-CVP	6 (12.5)
THP-CVP + R	4 (8.3)
Other regimens	6 (12.5)
Unknown	1 (2.0)
Outcome	
At 6 months (*n* = 45)	
Dead	(10/45) 22.2%
Alive	(35/45) 77.8%
At 1 year (*n* = 36)	
Dead	(14/36) 38.9%
Alive	(22/36) 61.1%

Abbreviations: CHOP: cyclophosphamide, doxorubicin, vincristine, prednisone; R: Rituximab; THP-CVP-pirarubicin, cyclophosphamide, vincristine, prednisone; EBV: Epstein-Barr virus, HCV: hepatitis C virus.

**Table 2:** Detailed clinical characteristics of 48 cases of HHV8-negative effusion lymphomas of B-cell lineage.

Case	Ref no.	Age/sex	Other disease	HIV	EBV	HCV	Sites involved	Morphology	Immunophenotype^#^	Molecular genetics/cytogenetics	Therapy	Outcome
1	Case 1	87/F	CHF, Afib	—	—	—	Pleura	Large	CD19, CD45, CD20, CD79a	*	Pleurodesis	Alive 21 mo
2	Case 2	82/F	HTN, sick sinus syndrome, COPD	—	—	—	Pleura	Large	CD20, bcl-6, MUM1/IRF4, PAX5	Clonal *IGH@ *	Pleural effusion drainage	Died 13 mo
3	[5]	99/F	*	—	—	—	Pleura, pericardium	Medium to large	CD19, CD20, CD5, CD25, IgM, IgD	*MYC* amplification but no rearrangement, Clonal *IGH@ *	Pleural drainage	Alive 16 mo
4	[5]	85/M	HTN, Afib	—	—	—	Pleura, pericardium	Medium to large	CD20	Clonal *IGH@, * no *MYC* rearrangement	No treatment	Alive 11 mo
5	[6]	79/M	HTN, CHF	—	—	—	Pleura	Large pleomorphic	CD45, CD20, CD79a, bcl-2, bcl-6, MUM1	Clonal *IGH@ *	Pleurodesis with doxycycline	Alive 55 mo
6	[7]	67/F	RA	—	—	—	Pericardium	Medium to large	CD20, CD79a	Clonal *IGH@ *	CHOP and then followed by MEPP, DEVIC	Died 16 mo
7	[8]	74/M	*	—	—	—	Pericardium	Medium to large	CD20	*	Rituximab + CHOP	Died 7 mo
8	[9]	63/M	DM	—	+	—	Peritoneum, pleura	Large pleomorphic	CD19, CD20, CD22, CD45, HLA-DR, bcl-2, kappa	Clonal *IGH@ *	CHOP	Died 5 mo
9	[10]	82/M	*	—	+	—	Pleura. pericardium	Medium to large	CD20, CD79a, Ig light chain restriction	*	CHOP	Alive 18 mo
10	[10]	73/M	*	—	—	—	Pleura. Pericardium, peritoneum	Large	CD20	*	CHOP	Alive 12 mo
11	[11]	77/M	Prostate ca, MI, idiopathic CD4+ T-cell lymphopenia	—	+	—	Pleura	Large	CD45, CD19, CD20, CD79a, CD38, CD71, CD30, lambda	Trisomy 18. No rearrangements involving *MYC, BCL2, BCL6, ALK* and *IGH *	CHOP	lost to follow up
12	[12]	68/M	*	—	—	—	Pleura	Large	CD20, CD79a	Clonal *IGH@*. No *MYC* rearrangement	R − CHOP	Alive 22 mo
13	[13]	78/M	Idiopathic CD4+ T-cell lymphopenia	—	+	—	Pleura, pericardium	Large	CD19, CD20, CD22, HLA-DR, IgM, bcl-6	Additional unknown material at 3q27 (*BCL6*). No *MYC* rearrangement	R + THP-COP	Alive 30 mo
14	[4]	88/M	CAD	—	—	—	Pleura	Large	CD20, CD30, CD79a, CD45	*	R + CHOP	Alive 11 mo
15	[14]	69/M	None	—	—	—	Pericardium, pleura	Large pleomorphic	CD19, CD20, CD5, kappa, bcl2, cyclin D1	t(8; 14) (q24; q32); *MYC-IGH* rearrangement. Clonal *IGH@ *	THP-COP	Died 5 mo
16	[15]	52/F	*	—	—	*	Pleura, pericardium	Large pleomorphic	CD19, CD20, CD22, CD45, HLA-DR	Clonal *IGH@ *	*	*
17	[16]	59/F	Hep C cirrhosis	—	−	+	Peritoneum	Small to medium-sized	CD20, CD10, IgG	48,XX,t(8;22)(q24;q11), +16, +21; *MYC-IGL* rearrangement. Clonal *IGH@ *	None	Died 2 mo
18	[17]	57/M	*	—	+	*	Peritoneum	Monomorphic, small to medium-sized	CD19, CD22, CD79a, CD10, CD23, CD38, IgM	46,XY,t(8;22)(q24; q11); *MYC-IGL* rearrangement	None	Died 1 w
19	[18]	63/M	Hep C cirrhosis, HCC	—	−	+	Peritoneum	Medium to large size	CD19, CD20, CD22, IgG lambda	Complex karyotype with t(9;14). No *MYC* rearrangement. Clonal *IGH@ *	None	Died 22 mo
20	[19]	60/F	Cholesteatoma	—	+	−	Peritoneum	Large	CD19, CD20, CD22, HLA-DR	Complex karyotype including der(8) t(2;8) (q31;q24), but no *MYC* rearrangement identified by Southern blot	None	Alive 24 mo
21	[20]	65/M	Hep C cirrhosis	—	−	+	Peritoneum	Large	CD19, CD20, CD22, IgH@	Clonal *IGH@*. No *MYC* rearrangement	Prednisolone, etoposide	Alive 8 mo
22	[21]	65/M	Alcoholic cirrhosis	—	+	−	Peritoneum	Large Immunoblastic	CD19, lambda	Clonal *IGH@ *	CHOP	Died 12 mo
23	[22]	75/M	*	—	−	−	Pleura	Large	CD19, CD20, HLA-DR, kappa	Complex karyotype including *MYC *amplification. Clonal *IGH@ *	CHOP	Died 15 mo
24	[22]	76/M	*	—	−	*	Pleura	Large	CD19, CD20, CD10, HLA-DR	Complex karyotype with t(8; 22)(q24,q11); Clonal *IGH@ *	None	Alive 6 mo
25	[22]	32/F	Congenital protein-losing enteropathy	—	−	*	Peritoneum	Large	CD19, CD20, CD10, HLA-DR	Complex karyotype including *MYC* amplification. Clonal *IGH@ *	CHOP, PBSCT	Alive 13 mo
26	[22]	81/M	*	—	—	*	Pleura	Large	CD19, CD20, CD10, HLA-DR, CD5	Complex karyotype including *MYC* amplification. Clonal *IGH@ *	None	Alive 2 mo
27	[23]	58/F	DM, Hep C, hypothyroidism	—	−	+	Peritoneum	Large	CD19, CD20, CD4, CD5	Hyperdiploid karyotype including *MYC* rearrangement. Clonal *IGH@ *	None	Died 7 mo
28	[24]	58/F	CVID	—	+	−	Pleura, pericardium	Large	CD19, CD20, CD22, HLA-DR, kappa	No *MYC* rearrangement	Prednisolone	Died 18day
29	[25]	58/M	Hep C cirrhosis	—	−	+	Peritoneum	Large	CD45, CD19, CD20, CD22, CD10, FMC7, HLA-DR	Clonal *IGH@ *	CVP	Died 5 mo
30	[26]	90/F	Afib	—	−	−	Pleura peritoneum, pericardium	Large	CD20, CD79a, bcl-2	*MYC* rearrangement. Clonal *IGH@ *	None	Died 5 mo
31	[27]	70/F	None	—	−	−	Pleura, pericardium	Large	CD19, CD20, CD22, CD24, CD8, CD10, HLA-DR, CD38	Complex karyotype. No *MYC* rearrangement. Clonal *IGH@ *	CHOP, Sobuzoxane	Alive 30 mo
32	[28]	32/F	Lymphangioma, protein-losing enteropathy, chylothorax, Hep C	—	−	+	Pleura, peritoneum	Large	CD19, CD20, CD10, HLA-DR	Complex karyotype including *MYC* amplification	THP-COP, PBSCT	Died 18 mo
33	[29]	74/F	Hep C cirrhosis, allergic granulo-matous angiitis	—	−	+	Pleura, pericardium, peritoneum	Large	CD45, CD19, CD20, CD25, HLA-DR, kappa	No *MYC* rearrangement. Clonal *IGH@ *	Rituximab + THP-COP	Alive 26 mo
34	[30]	75/F	−	—	−	−	Pericardium	Large	CD20, CD79a	t(1;22)(q21;q11), t(14;17)(q32;q23). No *MYC* rearrangement	CHOP	Alive 36 mo
35	[31]	90/M	History of TB	—	−	−	Pleura	Large	CD19, CD20, CD30	Complex karyotype including add(8)(q24). Clonal *IGH@ *	Rituximab + THP-COP	Alive 38 mo
36	[31]	87/F	*	—	−	−	Pleura	Large	CD20, CD30, kappa	*	Rituximab	Alive 32 mo
37	[32]	74/M	CKD, pulmonary infarction, DM	*	—	*	*	Large	CD19, CD20, MUM1, BLIMP1	Clonal *IGH@ *	Pleural effusion drainage	Died 80 mo
38	[32]	87/M	DM	*	—	*	*	Large immunoblasts type	CD19, CD20, MUM1	Clonal *IGH@ *	THP-CVP	Died 16 mo
39	[32]	66/M	DM, HTN, MI	*	—	*	*	Large	CD19, CD20, MUM1	Clonal *IGH@ *	THP CVP + rituximab	Alive 9 mo
40	[32]	94/F	Afib	*	—	*	*	Large	CD20	*	THP-CVP	Died 1 mo
41	[32]	92/M	CRF	*	—	—	*	Medium to large sized	CD19, CD20, bcl-6, MUM1	Clonal *IGH@ *	No chemotherapy initially; THP-CVP 4 mo later	Died 9 mo
42	[32]	79/M	DM, CRF	*	—	*	*	Large	CD19, CD20, MUM1	Clonal *IGH@ *	None	Alive 7 mo
43	[33]	76/F	Hypothyroidism, pulmonary emphysema	*	*	*	Pleura, pericardium	Medium-sized monomorphic cells	CD19, CD20, CD21, surface Ig, HLA-DR	X,Xq-,2q-,5q+,-6, +7p,+9p,+15,+r. Clonal *IGH@ *	Prednisolone	Died 15 mo
44	[34]	55/M	Autoimmune hemolytic anemia	—	+	—	Peritoneum	Large	CD45, CD20, CD79a, CD38, IgM	49,XY,add(3)(q11), der(8)t(1;8)(q12;p11),+r, +2mar. Clonal *IGH@ *	CHOP	Died 3 mo
45	[20]	65/F	Liver cirrhosis, Hep C	—	—	+	Peritoneum	Large	CD19, CD20, CD22	No *MYC* rearrangement	Prednisone, etoposide	Alive 8 mo
46	[35]	92/F	HTN, DM, ESRD	—	—	*	Pleura	Large	CD20, CD45, bcl-2	*	None	Died 2 mo
47	[36]	70/M	Hep B, liver transplant	—	+	—	Pleura	Large	CD19, CD20	*	None	Alive 8 mo
48	[37]	51/M	None	—	—	—	Scrotum	Medium to large size	CD45, CD19, CD20, CD79a	Clonal *IGH@ *	Carboplatin, etoposide, mitoxantrone, prednisone + radiotherapy	Alive 8 mo

^#^Immunophenotype includes only positively expressed antigens. *Information not available or mentioned. Abbreviations: Afib: atrial fibrillation; ca: carcinoma; CAD: coronary artery disease; CHF: congestive heart failure; CHOP: cyclophosphamide, daunorubicin, oncovin, prednisolone; CKD: chronic kidney disease; COPD: chronic obstructive pulmonary disease; CRF: chronic renal failure; CVID: common variable immune deficiency; DEVIC: dexamethasone, etoposide, ifosfamide and carboplatin; DM: diabetes mellitus; ESRD: end-stage renal disease; HCC: hepatocellular carcinoma; Hep: hepatitis; HTN: hypertension; *IGH@*: immunoglobulin heavy chain gene rearrangement study; MEPP: mitoxantrone hydrochloride, etoposide, cisplatin and prednisolone; MI: myocardial infarction; mo: months; PBSCT: peripheral blood stem cell transplantation; R: rituximab; RA: rheumatoid arthritis; TB: tuberculosis; THP-COP: pirarubicin, cyclophosphamide, oncovin, prednisolone
